# Is increasing inorganic fertilizer use for maize production in SSA a profitable proposition? Evidence from Nigeria

**DOI:** 10.1016/j.foodpol.2016.09.011

**Published:** 2017-02

**Authors:** Lenis Saweda O. Liverpool-Tasie, Bolarin T. Omonona, Awa Sanou, Wale O. Ogunleye

**Affiliations:** a Department of Agricultural, Food, and Resource Economics, Michigan State University, East Lansing, MI, United States; b Department of Agricultural Economics, University of Ibadan, Nigeria

## Abstract

Inorganic fertilizer use across Sub-Saharan Africa is generally considered to be low. Yet, the notion that fertilizer use is too low is predicated on the assumption that it is profitable to use rates higher than currently observed. There is, however, limited empirical evidence to support this. Using a nationally representative panel dataset, this paper empirically estimates the profitability of fertilizer use for maize production in Nigeria. We find that fertilizer use in Nigeria is not as low as conventional wisdom suggests. Low marginal physical product and high transportation costs significantly reduce the profitability of fertilizer use. Apart from reduced transportation costs, other constraints such as soil quality, timely access to the product, and availability of complementary inputs such as improved seeds, irrigation and credit, as well as good management practices are also necessary for sustained agricultural productivity improvements.

## 1 Introduction

Inorganic fertilizer use is considered low in Africa and many reasons have been cited to explain this. These include limited or untimely availability of the input (Carlsson et al., 2005, World Bank, 2006), imperfect markets (Abrar et al., 2004), lack of agronomic knowledge (Asfaw and Admassie, 2004), riskiness and credit constraints (Croppenstedt et al., 2003) and economies of scale in supply––which have all been invoked to give rise to “market smart subsidies”. While there are signs of an increase in fertilizer use, especially in those countries with subsidy programs (Nigeria, Malawi, and Zambia) or other concerted support (Ethiopia), fertilizer use generally remains low (Sheahan and Barrett, 2014, Sommer et al., 2013, Montpellier Panel, 2013, Banful et al., 2010).

Importantly, the belief that fertilizer use is too low is predicated on the assumption that it is profitable to use higher rates than is currently the case. However, there is little rigorous empirical evidence to support this notion. While various studies have explored the yield response of fertilizer in crop production, (Adedeji et al., 2014, Sommer et al., 2013, Omonona et al., 2012, Offodile et al., 2010, Akighir and Shabu, 2011, Xu et al., 2009), there are few studies that have actually explored the profitability of fertilizer use. Moreover, most studies on profitability are either outdated or largely based on case study areas––not nationally representative (Wopereis-Pura et al., 2002, Poussin et al., 2003, Becker and Johnson, 1999, Adedeji et al., 2014, Omonona et al., 2012).

Examination of the profitability of fertilizer use requires an understanding of: (1) fertilizer agronomics, (the yield response), and (2) fertilizer economics (the output/input price ratio as well as quantities and costs of inputs and transportation). This requires detailed information on agricultural practices and input costs. The Nigeria Living Standard Measurement Study-Integrated Survey on Agriculture (LSMS-ISA) dataset provides a unique opportunity to explore the profitability of fertilizer use in Nigeria. It is a nationally representative panel dataset rich with detailed agricultural information at the plot level. This makes it possible to specifically address the profitability of fertilizer use in a production function framework.

While various studies have explored the yield response of fertilizer in crop production, very few address the fact that there are likely unobserved characteristics that affect fertilizer application rates that also affect yields^1^ (Offodile et al., 2010, Akighir and Shabu, 2011, Adedeji et al., 2014). This paper uses the LSMS-ISA with plot level information to provide empirical evidence on the profitability of fertilizer use for maize production across Nigeria, addressing the endogeneity of the input use decision. We utilize panel data estimation techniques to estimate profit maximizing quantities of applied nitrogen for maize production across market conditions for the main cereal-root crop farming system that accounts for almost 65% of maize plots in the study sample. Then we compare these expected optimal rates to the actual rates used by maize farmers. We explore if fertilizer use pays at current prices and if not always, under which circumstances it does.

Thus, this paper addresses two gaps in the literature. First, we are able to more consistently identify the yield response to fertilizer application by accounting for unobserved time invariant household characteristics likely to affect fertilizer application and yields. Second, we are able to contribute to filling a key gap in the fertilizer use literature, which appears to believe fertilizer use is low in SSA, even though it is profitable. As a result of this assumption, the literature generally looks to other constraints to its adoption such as financial market imperfections, limited knowledge, lack of demand or economies of scale on the supply side (agro-dealer network), or lack of access to markets to sell the produce; but these all link again to profitability issues. This paper rather focusses on the profitability of fertilizer use as a likely explanatory factor for observed fertilizer use rates.

The rest of this paper is organized as follows: Section 2 describes fertilizer use generally, and within the major maize producing farming system in Nigeria within the context of prevailing food and fertilizer policies. Section 3 presents our conceptual framework and empirical methods. We present the production function estimates, marginal (and average) products of applied nitrogen, and the analysis of the profitability of nitrogen application for maize across various categorizations in Section 4. Section 5 concludes.

## 2 Fertilizer use across Nigeria

Since the 1940s, Nigerian governments have generally perceived that fertilizer use in the country was low. With the rising population density by the 1960s, the government became increasingly concerned about farmers’ awareness of fertilizer’s benefits (Whetham, 1966), and the effects of credit constraints (Ogunfowora and Norman, 1973). Since the 1970s, Nigerian governments have tried to stimulate fertilizer demand, grow the commercial fertilizer sector, and lower fertilizer prices. Strategies used to stimulate fertilizer use include subsidies, extension services to develop soil fertility management technologies, and programs to increase farmers’ access to credit. These programs, however, did not significantly raise fertilizer demand (Nagy and Edun, 2002).

**Fig. 1:**
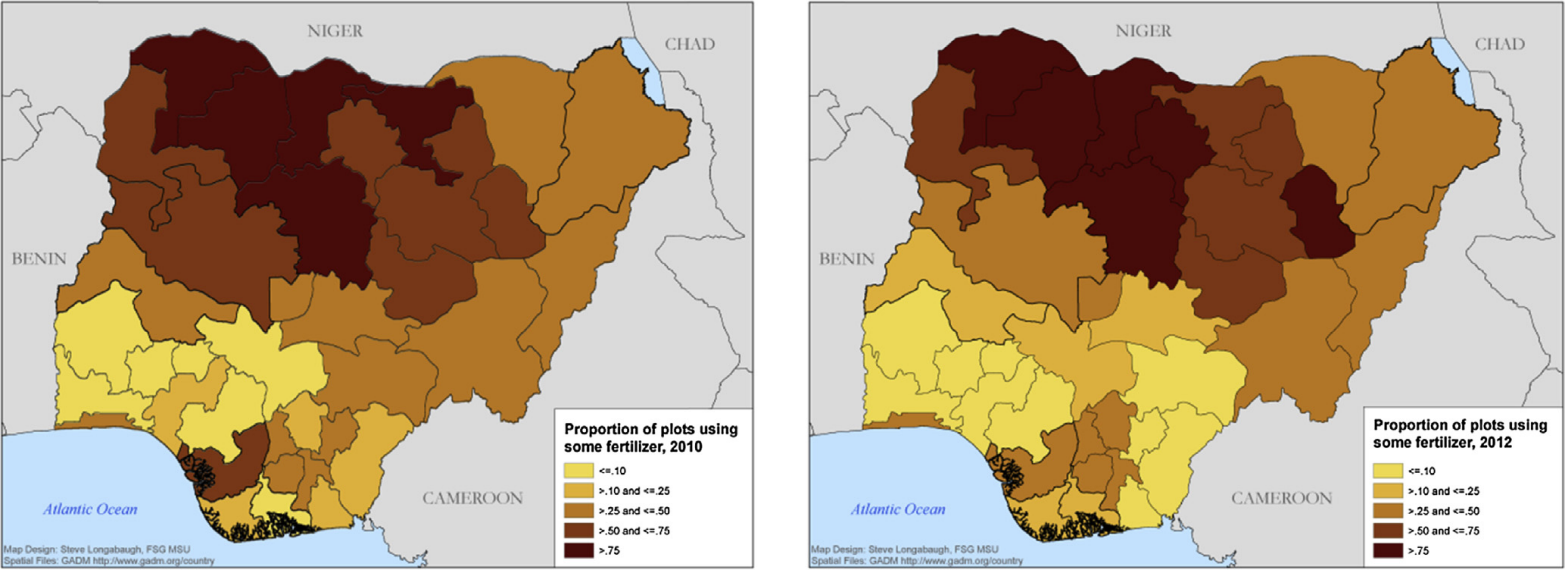
The proportion of plots on which inorganic fertilizer is applied in Nigeria (2010 and 2012).

Source: Data generated by authors from the 2010 and 2012 LSMS-ISA data. Map by Longabaugh (2014).

Despite the numerous factors cited as responsible for low fertilizer use, there is limited empirical evidence on the nature and rationale for the actual patterns of observed fertilizer use rates across Nigeria’s diverse farming systems and cropping patterns. Fertilizer use and needs will naturally vary depending on agro ecological and market conditions, government policies, cropping systems, and fertilizer responsiveness. Fertilizer use in the north is typically higher than in southern states (Fig. 1). This is partly attributed to lower soil fertility (FFD, 2011, Smith et al., 1997), larger area cultivated, and the growth of high value crops such as vegetables and particular cereals in the region (Eboh et al., 2006). Additionally, northern states have traditionally provided greater fertilizer subsidies since the colonial era when administrations provided support for fertilizer use out of concerns over soil depletion and desertification (Mustapha, 2003).

**Fig. 2:**
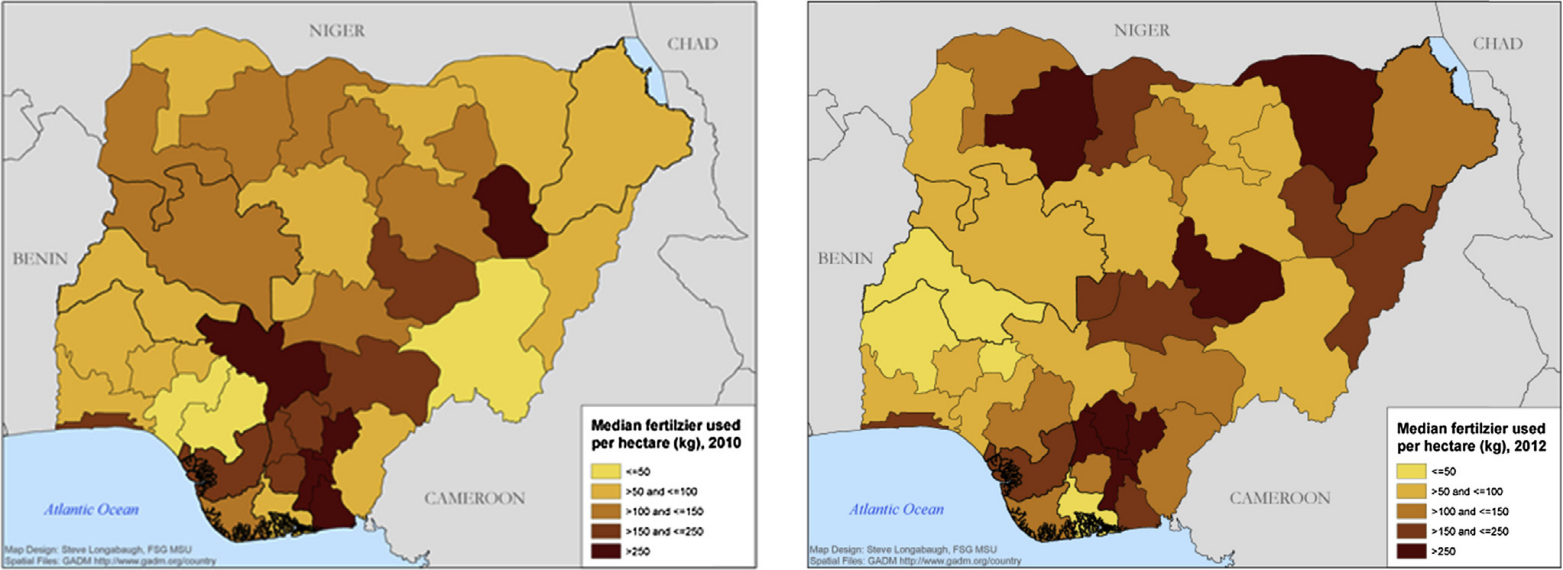
Median quantity of fertilizer applied per hectare of land in Nigeria (including zeros) 2010 and 2012.

Source: Data generated by authors from the 2010 and 2012 LSMS-ISA data. Map by Longabaugh (2014).

Contrary to conventional wisdom, Fig. 1 indicates that fertilizer use is quite common in Nigeria. Many Nigerian smallholder farmers use some inorganic fertilizer and in many states, some inorganic fertilizer is applied on over 70% of plots. Fertilizer use rates across all plots (including zeros) vary significantly across space and time and are often greater than 100 kg per hectare (Fig. 2). This is consistent with Sheahan and Barrett (2014) who find unconditional and conditional fertilizer use rates in Nigeria to be about 130 kg/ha and 310 kg/ha respectively.

### 2.1 Fertilizer use in maize production in Nigeria

This paper uses information extracted from the LSMS-ISA data for Nigeria. This dataset is nationally representative and includes detailed agricultural information collected at the plot and household level across Nigeria. The LSMS-ISA dataset includes geo-referenced plot locations and plot-level information on input use, cultivation, and production. The information was collected over two visits per household per year in 2010/2011 and again in 2012/2013. The first visit each year collected information on planting activities of the households, while the second collected information on post-harvest outcomes. For this analysis, we extract all plots on which maize was grown in the main agricultural season in each survey year. Thus, we have information on the size of maize plots, the amount of fertilizer and other inputs used, and the maize yields for over 1200 maize plots over two survey periods.

Maize is one of the three most important cereals grown in Nigeria along with sorghum and millet (USAID, 2010). Being a priority crop under the flagship agricultural programs of the Nigerian government since 2012, maize farmers have received intentional support in terms of access to subsidized fertilizer and improved seeds (Federal Ministry of Agriculture, 2011). Anecdotal evidence indicates that while farmers have enjoyed increased access to modern inputs with the current focus, issues of seed quality and low yields remain key challenges.

For this analysis, we focus on the main cereal producing area where we consistently have sufficient observations over time and robust results across model specifications. This subset accounts for over 60% of the plots in the study sample. Thus, while our results are not nationally representative, they can be considered representative of the main farming system for maize production in Nigeria. The cereal-root crop farming system (C-RCFS) found in the dry sub-humid agro ecological zone is characterized by relatively lower population density, higher temperatures, and lower altitude. Almost half (44%) of the maize plots in this farming system (in our sample) use animal draught and intercropping is relatively common and practiced on about 70% of maize plots.^2^

Table 1 reveals the extent and magnitude of fertilizer use for maize in the C-RCFS. Fertilizer use and application rates on average are similar over time, though there appears to have been a slight increase between 2010 and 2012. Furthermore, there does not appear to be a significant difference in yields between fertilizer users and the average sample. This likely reflects that there are other important factors explaining maize productivity and the effect of fertilizer use on maize yields besides fertilizer use. These could include the quality of the soil, input and output costs, the availability of fertilizer and other complementary inputs (such as water, seed, and organic manure), or other management practices.

## 3 Conceptual framework and empirical approach

Agricultural production is a key source of income for most rural households, alongside non-farm or off-farm activities. We assume that households decisions are made to optimize, not only over all these activities, but also at the plot level. Farmers need to decide the amount of risky inputs (such as fertilizer) to be applied on each plot. Modern inputs such as fertilizer typically increase both the mean and the variance of the net returns to production (Just and Pope, 1979). Generally, the amount of fertilizer used has to be decided before the rains have come or output price is known for sure, and in the presence of imperfect credit and insurance markets. Consequently, we follow previous work to consider the fertilizer use decision of a farmer as the solution to a constrained utility maximization problem as in Singh et al. (1986). This yields reduced form specifications of input demands and technologies, and output supply that are functions of input and output prices as well as various socio economic and household characteristics (Sadoulet and de Janvry, 1995). Once the decision on input use has been made, the effect of fertilizer use on maize yields on a farmers plot can be expressed with a production function as follows:

(1) $${\mathrm{Yield}}_{\mathrm{ijt}}=f(X_{\mathrm{kijt}}\text{,}Z_{\mathrm{hijt}}\text{,}u_{\mathrm{ijt}})$$

where ${\mathrm{Yield}}_{\mathrm{ijt}}$ refers to the yield per hectare (in kilograms) of maize on plot i for household j in time t which is a function of several vectors of endogenous **(*X*)** and exogenous factors (***Z***). $X_{\mathrm{kijt}}$, refers to a vector of plot and time specific determinants of maize yields, including the use of various inputs (including applied nitrogen) while $Z_{\mathrm{hijt}}$ is a vector of controls that affects crop production such as soil quality, access to information and markets as well as the level and distribution of rainfall (Tolk et al., 1999). $Z_{\mathrm{hijt}}$ also includes household characteristics including the age and gender of the plot manager, household wealth. Finally, $u_{\mathrm{ijt}}=\varepsilon_{\mathrm{ijt}}+c_{i}$ is a composite error term comprising time invariant ($c_{i}$) and time varying unobserved characteristics $\varepsilon_{\mathrm{ijt}}$ of our production system.

Our primary interest is in estimating the extent to which nitrogen use affects maize yields.^3^ Since most maize farmers in Nigeria typically use compound NPK as basal fertilizer or Urea alongside NPK as top dressing, there is a high correlation between applied nitrogen and phosphorus. Thus, the yield response of maize to applied nitrogen and phosphorous application cannot be assessed separately. Furthermore, while plants typically absorb the majority of applied nitrogen within the same season of application, the absorption process for phosphorus is much longer (Lanzer and Paris, 1981, Goedeken et al., 1988, Sheahan, 2012) making it difficult to accurately identify the yield response to applied versus previously existing phosphorous. For this reason (among others), studies on fertilizer yield response to cereals generally focus on nitrogen (Sheahan et al., 2013, Liverpool-Tasie, 2016; Ezui et al., 2010, Burke et al., 2014). This article also focusses on applied nitrogen while controlling for its interaction with phosphorous and noting the omitted variable bias this might introduce.^4^

One key challenge when estimating the effect of fertilizer on yields is the endogeneity of the decision to use fertilizer and the quantity of fertilizer applied on a maize plot. It is likely that fertilizer application is correlated with other farmer and plot specific characteristics (such as unobserved variation in soil characteristics, managerial skill, or ability) that are also likely to drive farmer yields and this restricts any causal interpretation to the coefficient on fertilizer use in a yield response model. This correlation between the unobserved individual effect in the error term $c_{i}$ and the rate of application of fertilizer would cause a bias in ordinary least squares (OLS) estimators (Hausman and Taylor, 1981). Consequently, our method of identification of the effects of fertilizer on yields is based on a household fixed effects model. The fixed effects method attenuates potential biases that can threaten our ability to consistently estimate the effects of fertilizer by using variation in fertilizer use within a household over time to identify the causal effect of fertilizer on yields (Wooldridge, 2010). While the fixed effects model addresses bias caused by time invariant factors (such as farmer ability that is crucial for production function estimates), it does not deal with any bias caused by time-varying unobservable factors that may be correlated with yields and correlated with the household’s fertilizer use such as plot and soil characteristics. One unique feature of this study is the availability of some plot level characteristics like plot elevation, slope and wetness potential that we include in our production function estimates. This addresses some of the usually absent, but important, time varying unobserved characteristics of concern when using fixed effects model in yield response estimations. While we focus on fertilizer, we recognize that most input use variables are likely endogenous and that the FE approach only addresses endogeneity due to time invariant unobserved household characteristics.

Another limitation of the fixed effects model is that we are unable to recover the coefficients on any time invariant observable characteristics as well. Given that our main concern is on fertilizer use, we do not consider this a major limitation.^5^ To confirm that our results are not biased due to the fact that we are not able to account for other unobserved plot characteristics that affect input use and yields, we also run a household fixed effects model at the household level. This household model focuses on the effect of total applied nitrogen (and other factors) on total household maize output. The main yield response findings are maintained and the household estimates are presented in Table A1 in the appendix.^6^ We use a modified quadratic production function for this analysis and, thus, do not include all possible interactions. We include the typical variables used in other studies in Sub-Saharan Africa with due consideration for the Nigerian context based on studies from the country (Onuk et al., 2010, Gani and Omonona, 2009, Kehinde et al., 2012) and to maximize available degrees of freedom.

To address challenges associated with extreme outliers, both the input and output variables were winsorized at 99% (or 95% where values at 99% still seemed very large). This involves replacing extreme outlier values beyond the 99th percentile with the value at the 99th percentile rather than dropping the variable. However, where fertilizer use per hectare was still larger than 1 ton after winsorizing, such observations were replaced with a cap value of 700 kg per hectare.^7^ Due to challenges associated with using the labor data for the first wave of data, household adult equivalency units and the use of hired labor on a plot were used as proxies for labor used.^8^ Due to challenges associated with the units of measures of the quantity of herbicides and pesticides used by farmers, we use a dummy to account for whether a farmer uses a chemical (herbicide or pesticide).

To control for the fact that improved seed varieties are often a complementary input to inorganic fertilizer, we include whether seed used was commercially purchased. This assumes that most improved seed is hybrid that needs to be purchased each year and not open pollinated varieties. We also include measures of the plot’s slope (measured in degrees), plot elevation measured in meters above sea, the tropical wetness index/plot wetness potential index and length of growing period (Wilson et al., 2007). A dummy variable is used to distinguish farmers who planted maize as a sole crop on the plot, versus those engaged in intercropping. While mono cropping could be a sign of specialization in maize production for commercial purposes, intercropping of crops such as maize with legumes is also commonly used to diversify risk and increase maize yields because of the nitrogen fixing effect of legumes. As a source of additional nutrients likely to affect maize yields as well as response of nitrogen application, we control for organic manure use and the number of other crops grown on the plot. While growing more crops on a plot might indicate competition for nutrients, the kind of crops grown (e.g., if they are leguminous crops that fix nitrogen) could also indicate differential effects of applied nitrogen and consequent maize yields.^9^ We also control for plot ownership defined as whether the plot was purchased or distributed by community or family.^10^ Finally, where possible, we control for the geopolitical zones to account for any region specific characters or policies that could affect maize yields. In all specifications, standard errors are clustered at the household level to make them robust to serial correlation and to account for non-constant variance (Wooldridge, 2010). Since our analysis is focused on the balanced panel of households that were present in both waves and we don’t account for movers and split offs there is the potential for attrition bias. We test for it using the regression-based approach (ibid.). We fail to reject the null hypothesis of no attrition bias (p > 0.34) for our production function estimation.

## 4 Production function results

Our descriptive statistics indicate that maize production is largely a smallholder activity in Nigeria. The average maize plot is between 1 and 1.5 ha, managed by a middle-aged male with limited use of irrigation and mechanization. While only about 20% of maize plots use purchased seed, almost 50% of farmers use some chemicals (herbicides and pesticides) in maize production; the average fertilizer use is between 40 and 45 kg of applied nitrogen This figure is not conditional on use (e.g., Table 1) and translates to between 150 kg and 170 kg/ha of fertilizer.^11^ This is almost identical with Sheahan and Barrett (2014) who find unconditional fertilizer use for Nigeria to be about 130 kg/ha using the 2010/11 data. As Table 2 suggests, the average nitrogen application per hectare (as well as most input use variables) among maize farmers is relatively consistent across years lending credibility to the data. Maize prices vary widely across the different states of Nigeria, which likely reflects state level differences such as proximity to the port (for fertilizer), local consumption and production of maize. Average state level prices generally have a standard deviation less than 15.

### 4.1 Production function estimates and marginal physical product of nitrogen

The production function estimates are presented in Table 3. The negative squared terms imply decreasing returns to applied nitrogen and indicate that the quadratic functional form used is likely appropriate. We do not find a positive effect of improved seed use in the presence of applied nitrogen.^12^ The general insignificance (and negative sign) might reflect the poor quality of commercially purchased seed; often a problem in Nigeria (Ajeigbe et al., 2008). The seeding rate appears to be a major determinant of maize yields in Nigeria. Higher labor supply, the use of mechanical equipment, chemicals, and organic manure tend to increase maize yields in the main farming system.^13^ The positive effect of organic manure use likely indicates the importance of the soil organic matter content for maize yields given the often low reserves of inherent nutrients in Nigerian soils. This supports the findings of several studies on the importance of soil quality for the yield response of applied nitrogen (Marenya and Barrett, 2009, Snapp et al., 2014). Where significant, maize plots on which fewer crops were grown (apart from maize) had better yields than those on which crops other than maize were grown. This likely indicates that while mixed cropping (e.g., with a leguminous crop) could improve soil nutrient content (and consequently fertilizer application), thus, yields, additional crops also lead to more competition for nutrients.^14^

Maize production in Nigeria appears to exhibit the inverse relationship between farm size and physical yield. The plot size variable and its square are negative and positive respectively with both coefficients significant at 1%. This corresponds with a similar study on rice in Nigeria and several other studies sustaining the long debate on this relationship (Chayanov, 1966, Sen, 1962, Berry and Cline, 1979, Barrett, 1996). Table 3 also shows the importance of addressing the effects of unobserved household specific characteristics when estimating nitrogen yield response functions. The difference between the pooled OLS and fixed effects results indicate the importance of accounting for time invariant unobserved factors that are likely correlated with nitrogen application as well maize yields.

### 4.2 MPPs and APPs of applied nitrogen in Nigeria

The marginal physical products (MPP) for applied nitrogen in Nigeria are quite low (Table 4). The average MPP of nitrogen in main production in the main farming system where almost 65% of maize production in our sample occurs is about 7.5 kg. Though usually focused on a very specific location, other studies in Nigeria reflect these relatively low fertilizer yield responses Onuk et al. (2010) and Gani and Omonona (2009) find MPP values about 2 while Kehinde et al. (2012) found negative APP values. Studies on fertilizer yield response in Mfantseman Municipality in the Central Region of Ghana yielded an MPP value of 0.12. This is much lower than the potential yields of up to 50 kg maize per kg nitrogen when researcher management protocols are followed (Snapp et al., 2014). It is also quite different from what has been found in eastern and southern Africa. Sheahan et al. (2013) estimate an overall MPP of nitrogen for maize production to be about 17 (though this varies across space and time). Matsumoto and Yamano (2011) found marginal products ranging between 11 and 20 across the western and higher potential regions of Kenya while Marenya and Barrett (2009) found the marginal product of nitrogen to be 17.6 for Vihiga district (Western Province) of Kenya. The low MPPs of applied nitrogen in maize production indicate that increasing fertilizer use alone might not be sufficient to increase maize yields to desired levels in Nigeria, as a low maize yield response to nitrogen application is likely to significantly affect the profitability of its use.

## 5 Profitability of applied nitrogen for maize production

**Fig. 3:**
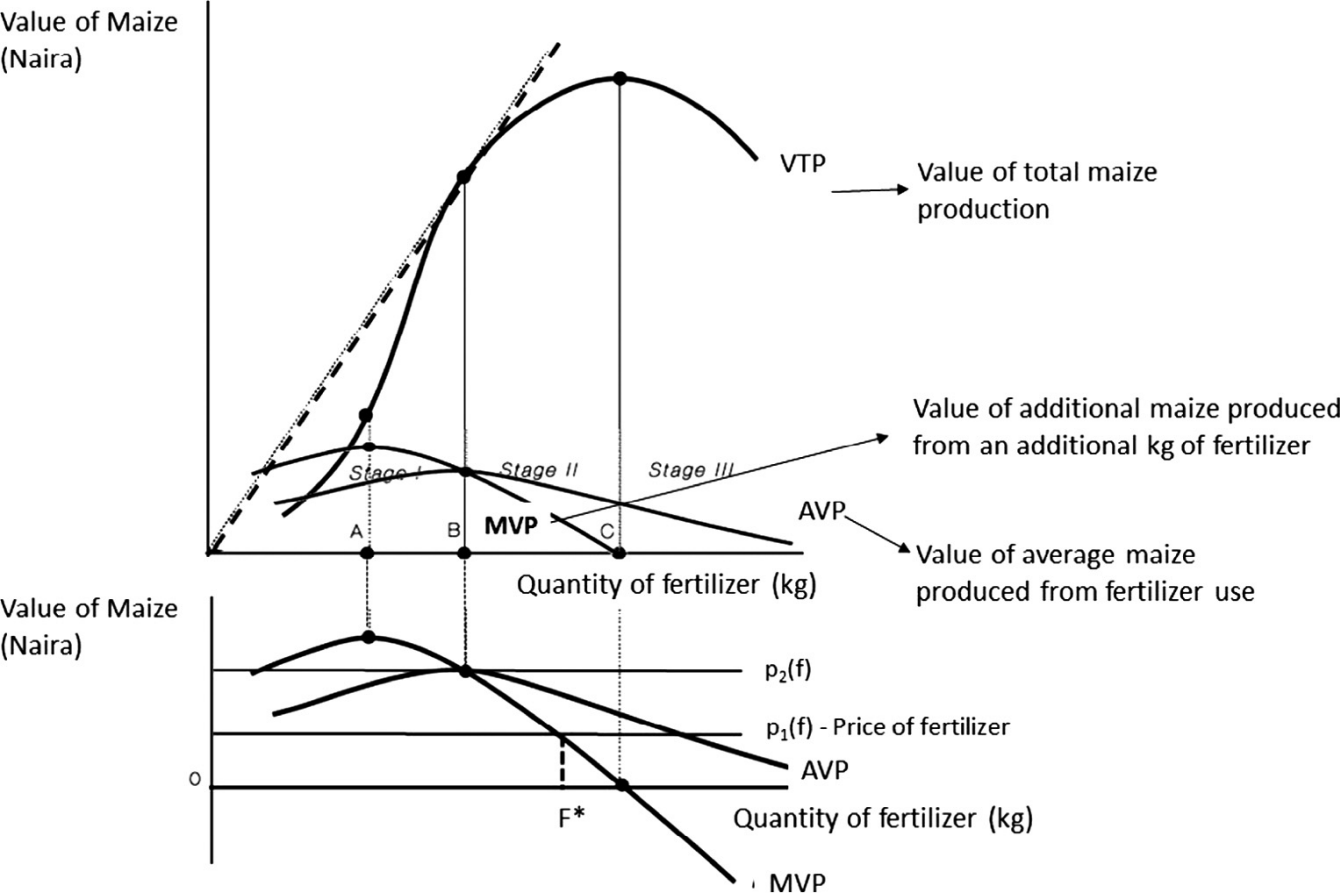
The optimal quantity of fertilizer for a profit maximizing (risk neutral) farmer.

Source: Authors, based on Debertin (2002: 53). Note that the output price is held constant.

Based on microeconomic principles, a risk-neutral, profit-maximizing farmer will find it profitable to use fertilizer if the value of the average kg of maize produced per kg of fertilizer (i.e., the average value product, AVP) is higher than the per kg price of fertilizer. For example, in Fig. 3, at any fertilizer price above p_2_(f), it would not be profitable to use fertilizer because the value of the average maize produced from fertilizer use would not cover the cost of that fertilizer. However, the *quantity* of fertilizer the farmer will use in order to maximize his/her profits will be determined by the price of the fertilizer, p(f) that is equal to the value of the additional maize produced from that unit of fertilizer (i.e., the marginal value product, MVP). In Fig. 3, the profit-maximizing quantity of fertilizer would be F^∗^. If a farmer uses more than F^∗^, s/he would reduce overall profit, since the additional maize produced from using an additional kg of fertilizer (beyond F^∗^) would not cover the cost of that fertilizer. Even so, fertilizer use would still be profitable though, as long as AVP > p(f).

For our profitability analysis, we use our fixed effects production function estimates to determine the profitability of fertilizer use. We calculate the MPP of applied nitrogen (which describes how much extra maize output can be produced by using one additional unit of applied nitrogen, all else held constant) by taking the first derivative of the production function with respect to applied nitrogen as in (2)

(2) $$\mathrm{MPP}=\frac{\partial ({\mathrm{Yield}}_{i\text{,}j\text{,}t})}{\partial ({\mathrm{Nitrogen}}_{i\text{,}j\text{,}t})}=K+\beta_{i}{\mathrm{Nitrogen}}_{\mathrm{ijt}}*X_{\mathrm{kijt}}$$

where K is the coefficient on the applied nitrogen variable and the $\beta_{i}$’s are the coefficient on the interaction terms between applied nitrogen and other plot and household characteristics. We conceptualize and calculate the average physical product as the gain in maize yield per unit of applied nitrogen relative to not using any applied nitrogen (Sheahan et al., 2013). These MPPs and APPs are then used to calculate our partial profitability measures; the Average value cost ratio (AVCR) and the marginal value cost ratio (MVCR) as follows:

(3) $$({\mathrm{AVCR}}_{\mathrm{nijt}})=\frac{\left(P_{\mathrm{mtv}}*{\mathrm{APP}}_{\mathrm{nijt}}\right)}{p_{\mathrm{nijt}}}$$

(4) $$({\mathrm{MVCR}}_{\mathrm{nijt}})=\frac{\left(P_{\mathrm{mtv}}*{\mathrm{MPP}}_{\mathrm{nijt}}\right)}{p_{\mathrm{nijt}}}$$

where ${\text{p}}_{\text{nijt}}$ is the acquisition price of nitrogen (market price for nitrogen plus transportation cost) and ${\text{p}}_{\text{mtv}}$ is the price of maize in the farmers community (v). From (3) when the value of the average quantity of maize ($P_{\mathrm{mt}}*{\mathrm{APP}}_{\mathrm{nijt}})$ is equal to the price of nitrogen $\left({\text{p}}_{\text{n}}\right)$ the ${\mathrm{AVCR}}_{\mathrm{nijt}}$ will be 1 and when the ${\text{AVCR}}_{\text{nijt}}$ is greater than or equal to one, the net benefit from using fertilizer is positive for a risk neutral household and it is profitable to use fertilizer. Similarly, from (4), when the value of the additional maize that can be produced with a unit of fertilizer is equal to its price, the MVCR will be equal to 1. When ${\text{MVCR}}_{\text{nijt}}$ is greater than 1, it implies that a risk neutral household could increase its income by increasing its nitrogen application rate as the current rate is not profit maximizing. Similarly a farmer could increase profits by reducing nitrogen application rates when ${\text{MVCR}}_{\text{nijt}}<1$.

As mentioned above, fertilizer use is risky and rural households in Nigeria are likely to be risk averse. Consequently while we focus on the risk neutral case, we present the distribution of MVCRs and recognize that the threshold of 1 (assuming risk neutrality) likely overestimates the estimated level of nitrogen that a profit-maximizing farmer would be expected to use if he were risk averse.

The output price used for this analysis was the median community selling price of maize per kilogram. While it is likely that a farmer’s decision to use fertilizer during the planting season is driven by expected prices of maize rather than the actual price at post planting or post-harvest, the unavailability of good price information at the community or local government area (LGA)^15^ level precluded our ability to explore options to generate such expected prices as described in Muyanga (2013) and used by Sheahan et al. (2013). By using the selling price we are assuming farmers had a good sense of those prices at planting time. We replace missing maize price values with local government medians and then state medians when LGA medians are unavailable.

We consider both the market price for nitrogen and the acquisition price (market price plus transportation costs). The market price used for nitrogen is a simple average of the market price of the nitrogen components of Urea and NPK converted to a one-kilogram equivalent (Xu, 2008, Sheahan et al., 2016). The market price for nitrogen was calculated as the value paid for fertilizer divided by the quantity purchased adjusted for the nutrient quantity. Where the resulting price of fertilizer was missing, the local government average (obtained from the data) was used; where that was not available, a state level average fertilizer price was used. Extreme values (greater than N250/kg) were also replaced with the local government or state average. Based on data collected from the Nigerian agricultural markets information service (gathered by the Federal Ministry of Agriculture and Rural Development), the price for nitrogen in 2010 generally ranged between N195 and N260 per kg of nitrogen from Urea fertilizer (with 46% nitrogen) and between N600 and N800 per kg of nitrogen from NPK (15% nitrogen).

With few community level input suppliers and poor infrastructure in rural areas, market prices do not always adequately reflect the cost of acquiring fertilizer. Transactions cost more generally (and transport costs more particularly) have been shown to play a key role in farmers’ decisions to use modern inputs (Winter-Nelson and Temu, 2005, Morris et al., 2007, de Janvry et al., 1991, Key et al., 2000). We thus calculate the fertilizer acquisition price as the market price for nitrogen plus the cost of transporting the nitrogen from the market to the farm. The most common mode of transporting fertilizer in our study sample was a motorcycle. The average transportation cost borne by farmers (based on farmer responses) to get their fertilizer was about N380 a trip and the average quantity of nitrogen nutrients purchased in a trip is about 23 kgs of nitrogen.^16^ For each farmer we calculate the average transportation cost per kg of fertilizer purchased as the total cost (in naira) for the trip divided by the quantity of fertilizer purchased (in kg). The survey question gives us the transportation costs borne by a farmer for the purchased fertilizer used on a plot. We add this average transportation cost per kg of fertilizer used on a plot (that was purchased) to the market price (per kg) of fertilizer to get the acquisition price (per kg) of fertilizer. For both the market price and transportation cost, the nitrogen equivalent of fertilizer is calculated as described earlier. The average price of nitrogen (per kg) in the C-RCFS was about N330 in 2010 and N320 in 2012 while the average acquisition price paid by farmers in our sample was about N370and N375 for both years respectively.^17^ This is about a 12%–18% increase in the cost of using fertilizer due to transportation cost in each survey year.^18^ This echoes the findings of other studies that showed high transportation costs in Nigeria; accounting for up to 20–25% of the urban retail prices at regional hub cities (Liverpool-Tasie and Takeshima, 2013). This effect is likely exacerbated at rural markets and remote villages. Other assumptions about the transportation cost calculation could yield higher transportation cost effects on the acquisition cost for rural farmers. See Sheahan et al. (2013) and Liverpool-Tasie (2016) for some alternative assumptions.^19^ High transportation costs were similarly observed in rural Ethiopia where Minten et al. (2013) found that farmers living about 10 km away from a distribution center faced transaction and transportation costs (per unit) that were as large as the costs needed to bring fertilizer over about a 1000 km distance from the international port to the input distribution center.

### 5.1 Profitability of fertilizer use for maize production in Nigeria

With the low MPP of nitrogen for maize production for a majority of farmers in Nigeria, the percentage of maize plots for which the net benefit from nitrogen application is positive (for a risk neutral) at the observed fertilizer acquisition prices and maize price is about 50% and 55% (i.e., plots for which the ${\text{AVCR}}_{\text{n}}>1$) in the main C-RCFS in 2010 and 2012 respectively (see Table 5). It also appears that about 50% of maize plots could increase their income by expanding nitrogen application (MVCR > 1) in both years. This implies that most of the farmers who could profitably use fertilizer are actually using less than optimal amounts. It should be noted that once risk preferences are taken into consideration, this percentage will be even lower as the profitability has to be higher to induce risk averse farmers to use fertilizer.

To explore the effect of transportation cost on the profitability of nitrogen application, we recalculate the AVCRS and MVCRs of fertilizer use at the plot level if fertilizer was available in a farmer’s community (transport cost = 0). In this case the net benefit from fertilizer use would be positive for almost 90% of the study plots (based on AVCRs) and over 70% of the plots could actually increase their income through expanding their use of fertilizer (Table 6). This demonstrates the importance of recognizing that the costs incurred to use modern inputs often extends significantly beyond the market price. When the market price alone is used to evaluate profitability (assuming zero transport cost), it would appear that fertilizer use is more profitable than in reality. This study only focusses on transportation costs but there are other transactions costs associated with securing modern inputs (multiple trips to the market and or arrangements necessary to identify where the input is available).

From Eqs. (3), (4) (and Fig. 3), another way to increase the profitability of fertilizer use would be to reduce the price of fertilizer. One way this could happen is the use of fertilizer subsidies. Fertilizer subsidies have been a dominant component of agricultural input programs throughout most of Nigeria’s recent history. It accounts for substantial shares of government capital spending on agriculture (Mogues et al., 2012). Under the current scheme in Nigeria, participating farmers receive two bags of subsidized fertilizer (typically subsidized at 50% of market price), in contrast with the immediate past program where no quota existed and subsidy levels between 25% and 75% was possible.^20^ In the event that majority of maize farmers could receive subsidized fertilizer this could significantly reduce the cost of fertilizer use. Recent empirical evidence on national fertilizer subsidy programs suggests that it is typically larger and more affluent farmers that benefit from such programs (Mason and Jayne, 2013, Ricker-Gilbert et al., 2011). Where not properly targeted, subsidy programs can crowd out commercial fertilizer purchases (Mason and Jayne, 2013; Ricker-Gilbert et al., 2011, Takeshima and Nkonya, 2014) Furthermore, given that less than 20% of applied fertilizer in Nigeria is likely to be subsidized (Takeshima and Liverpool-Tasie, 2015), the relative costs and benefits of such a strategy should be carefully considered.

Yet another way that the profitability of fertilizer use could be increased (from Fig. 3 and Eqs. (3), (4)) would be to increase the yield response of applied nitrogen on maize production (MPP). This could be achieved through complementary practices such as irrigation facilities, good quality seed, and other more efficient methods of fertilizer use or crop management practices. Though our results do not reveal strong complementarity effects this might reflect the limited use of complementary inputs on the same plot as demonstrated by Sheahan and Barrett (2014). However, given the potential benefit, this should be explored. Issues of soil organic content and other properties likely to increase the efficiency of applied nitrogen use ought to also be explored (Marenya and Barrett, 2009). Two key soil fertility constraints in many regions of Nigeria and other West African countries are low reserves of inherent nutrients and soil acidification due to continuous cultivation (Jones and Wild, 1975). While the application of inorganic fertilizer can address this constraint, the efficiency of these inorganic fertilizers is typically low on depleted soils, since soil organic matter helps to hold on to nutrients (that would otherwise be lost through leaching and runoff) later released to crops when needed. Similarly, the soil pH (potential hydrogen) level is key for efficient absorption of nutrients in inorganic fertilizers. Merely applying inorganic fertilizer can result in fertilizer wastage of up to 70% for extremely acidic soils with pH level of 4.5 or below (The Mosaic, 2013).

### 5.2 Fertilizer profitability and observed use rates

Next, we compare actual observed fertilizer use rates on maize plots in Nigeria with the expected profit maximizing levels. Following Sheahan et al. (2013) we use the estimates from the production function to derive the amount of nitrogen that should be applied for the MVCR to be equal to 1 for a risk neutral famer given the acquisition price of fertilizer and the market price. We rely on the concavity of the production function and the interaction between nitrogen and other variables to get plot level MPPs, AVCRs and MVCRs.

We see that the percentage of plots on which nitrogen is actually applied in the C-RCFS is higher than what would be expected at the current costs and yield response of fertilizer. While the net benefit from fertilizer use is only positive for about 50% of plots when risk neutrality is assumed, we have over 65% of maize plots using some fertilizer (Table 7). This likely indicates that fertilizer use is not purely driven by observed market prices and MPP. For example, for food security concerns, (particularly when faced with poor quality soils), the shadow price of maize might be much higher than the observed market prices. Some of these factors might be correlated with the true decision prices for farmers not observed in our study.

The average estimated optimal nitrogen application rates varies significantly across the maize plots in our sample in each survey year and is over 80 kg per hectare. These high optimal rates are partly driven by the low MPP values used in their calculation. When we look at individual plots, application rates are actually higher than desirable for between about 15% and 25% of current fertilizer users with an average gap of between 10 and 15 kg (across all plots^21^). In the absence of information about subjective expectations about rainfall and yields, these results suggest that there are a good number of maize farmers in Nigeria whose nitrogen application rates are higher than what one would expect. It appears that while expanding nitrogen application for maize production in Nigeria is necessary for some, quite a few farmers (for which fertilizer use is profitable) could potentially reduce their application levels. While high transportation costs are partly responsible for the limited profitability of nitrogen application for maize production, the low MPP of applied nitrogen for maize in Nigeria is also a key factor.

## 6 Conclusions

This paper looked at the effect of nitrogen application on maize production across the main maize farming system in Nigeria. Mitigating the effect of potential endogeneity of nitrogen application when estimating a maize production function, we find that the MPP of nitrogen is quite low in Nigeria. We also find that the expected profitability of nitrogen application for maize production is low for many farmers. Though this is partly driven by the relatively low MPP of nitrogen, high transportation costs are another factor that significantly reduces the profitability of nitrogen application.

We find that there is significant scope for increasing profitability through reduced transportation costs. While benefits of improvements in infrastructure and access to fertilizer (at the community level) are more universally spread among rural farmers relative to programs like fertilizer subsidies, this cost reduction could also be achieved through programs that encourage the setup of retail depots within communities or in smaller towns closer to farmers. Though the market price may increase, it will likely still be cheaper to transport fertilizer in bulk closer to many farmers (say in a state in Nigeria) than the cost that many farmers would have to bear to individually travel 40–70 km to a fertilizer distributor. Innovative schemes by the private sector which use industrious farmers within communities to serve as village promoters (teaching farmers about new technologies and selling inputs) could further reduce the distance farmers have to travel and, hence, transportation costs, and increase the expected profitability of fertilizer use for many rural farmers (Liverpool-Tasie et al., 2015). Significant reforms are underway in the Nigerian agricultural sector, particularly with regards to fertilizer. These reforms might change these results and, thus, should be studied.

In addition to reducing the distance farmers have to go to secure fertilizer (transportation costs), improving the yield response to nitrogen in Nigeria is key for the profitability of fertilizer use. In addition to the likely gains from complementary input use and improved management practices, more attention likely needs to be paid to understanding and addressing soil health. Understanding the soil organic matter and soil chemical properties is very important and likely necessary for any increased use of fertilizer in Nigeria to translate to meaningful increase in farmer productivity. This will also likely increase the effectiveness of subsidy programs that increase farmer access to inorganic fertilizer.

Generally, this study confirms that fertilizer use, which is clearly evident in maize production in Nigeria, can be profitable.^22^ However, at current MPPs, input and output prices, this remains a reality for only a subset of maize farmers. We find that some maize farmers in Nigeria are already applying nitrogen beyond levels considered economically optimal. This indicates the need for further studies on fertilizer profitability in Sub-Saharan Africa to understand why. This study only focusses on maize, but indicates issues that are likely to affect fertilizer use for other crops. See Liverpool-Tasie (2016) for the case of rice. More effort is needed to understand the rational for the current nitrogen application rates across smallholder farmers and to increase the profitability of fertilizer use by addressing transportation costs and other factors (such as soil quality, timeliness of availability, and management practice) currently mitigating the yield and profitability effects of fertilizer use.

## Figures and Tables

**Table A1:** Household level fixed effects results at the household level for the C_RCFS.

Household maize production	Pooled OLS	Fixed effects
Total nitrogen used	5.961⁎	7.835⁎
Total nitrogen squared	−0.010⁎	−0.013+
Nitrogen ∗ phosphorus	0.017⁎⁎	0.018
Total quantity of seeds used	1.198	1.165
Labor (adult equivalency unite)	4.158	42.872
Hired labor	161.083	186.919
Mechanization (1/0)	580.042	475.083
Irrigation (1/0)	126.321	91.965
Animal traction use (1/0)	378.483⁎	353.349
Chemicals (1/0)	166.816	197.121
Organic fertilizer (1/0)	350.038⁎⁎	25.638
Commercial seed	5.947	3.343
Commercial seed ∗ nitrogen	−1.665	−1.051
Male (1/0)	956.393⁎⁎	561.163
Age (years)	2.351	15.143
Assets (“000 Naira)	0.027	0.083
Plot area (hectares)	−130.364	−299.791
Squared plot area (hectare)	2.290	28.007
Topographic wetness index (units)	−17.143	−133.668
No other crop planted	985.766⁎⁎	1,268.598+
One other crop planted	617.145+	1,503.806⁎
Two other crops planted	200.792	1,116.263
Three other crops planted	−43.928	955.386
Year	322.671⁎	286.435
Number of observations	691	691
R-squared	0.11	0.64

Source: Authors’ estimations from the LSMS-ISA data.

^∗∗∗^Significant at 1 percent.

+ Significant at 15 percent.

⁎ Significant at 10 percent.

⁎⁎ Significant at 5 percent.

**Table 1:** Fertilizer use rates and maize yield in the main cereal root crop farming system.

	2010	2012
Mean fertilizer use per hectare^a^	197.8	211.0
Proportion of plots using fertilizer	0.64	0.67
Mean maize output per hectare (kilograms)	1054	1256
Mean maize output per hectare for fertilizer users (kilograms)	1115	1332
Number of observations (plots)	584	637

Source: Authors’ estimations from the LSMS-ISA data.

a These mean values are conditional on use.

**Table 2:** Descriptive statistics for key study variables.

Variables	2010		2012	
	Mean	Std. dev.	Mean	Std. dev.
Household adult equivalency units (units)	5.88	2.74	6.27	2.99
Male plot manager (1/0)	0.95	0.21	0.96	0.20
Hired labor (1/0)	0.50	0.50	0.49	0.50
Any household member could sell land (1/0)	0.78	0.42	0.80	0.40
Age of plot manager (years)	47.18	13.86	47.18	13.89
Owned household assets (Thousand Naira)	136.83	242.81	101.78	155.26
Area planted (hectares)	1.35	1.69	1.23	1.27
Nitrogen applied (kilograms per hectare)	40.19	43.75	46.56	68.36
Phosphorus per hectare (kilograms)	15.05	25.90	15.77	24.67
Seeding rate (kilograms per hectare)	19.20	19.59	22.60	22.92
Organic fertilizer (1/0)	0.02	0.15	0.02	0.15
Farmer purchased seed (1/0)	0.15	0.36	0.13	0.33
Mechanization (1/0)	0.07	0.26	0.04	0.20
Animal traction use (1/0)	0.45	0.50	0.44	0.50
Irrigation (1/0)	0.02	0.14	0.02	0.14
Agro chemical use (1/0)	0.42	0.49	0.48	0.50
Topographic wetness index (units)	14.14	1.87	14.13	1.79
Slope (percent)	3.73	2.88	3.50	2.79
Annual mean temperature (°C∗10)	297.80	14.65		
Annual precipitation (mm)	1120.00	187.50	1077.39	188.04
Plot elevation (meters)	526.33	261.47	554.86	289.81
No other crop planted (1/0)	0.29	0.46	0.21	0.41
One other crops planted (1/0)	0.43	0.50	0.45	0.50
Two other crops planted (1/0)	0.17	0.31	0.23	0.42
Three or more other crops planted (1/0)	0.09	0.30	0.11	0.31
Legume grown on plot (1/0)	0.29	0.45	0.29	0.45
Maize price (Naira per kilograms)	78.01	55.44	98.78	67.86
Fertilizer price (Naira per kilograms)	106.03	44.05	98.29	40.70

Source: Authors’ calculations using LSMS-ISA data (2010/2011 and 2012/2013). All prices are adjusted to 2012 prices using the cpi from the Nigerian National Bureau of Statistics.

**Table 3:** Production function estimates.

Maize yield on plot	Pooled OLS	Fixed effects
Nitrogen	4.688⁎	8.597⁎⁎
Nitrogen squared	−0.001	−0.004
Nitrogen ∗ phosphorus	0.024	0.021
Seed rate (kg/hectare)	12.849⁎⁎⁎	14.056⁎⁎⁎
Labor (adult equivalency unite)	3.958	171.032
Hired labor	197.446⁎	338.300⁎
Mechanization (1/0)	22.751⁎	64.576+
Irrigation (1/0)	397.193	8.706
Animal traction use (1/0)	183.212	289.753
Chemicals (1/0)	168.271	62.892⁎
Organic fertilizer (1/0)	389.337⁎	268.881+
Commercial seed	270.849	137.064
Commercial seed ∗ nitrogen	−4.300⁎⁎	−1.375
Male (1/0)	686.729⁎⁎⁎	123.725
Age (years)	6.182⁎	8.793
Assets (“000 Naira)	−0.050	0.001
Plot area (hectares)	−675.652⁎⁎⁎	−603.234⁎⁎⁎
Squared plot area (hectare)	71.071⁎⁎⁎	61.075⁎⁎⁎
Topographic wetness index (units)	−56.676⁎	−30.409
No other crop planted	227.909⁎	106.341⁎
One other crop planted	261.100	270.064
Two other crops planted	−20.229	236.743
Plot elevation (meters)	0.556⁎	0.691
Slope (percent)	−13.885	−48.229
Annual precipitation (mm)	0.111	−4.349
Any household member could sell land (1/0)	−54.520	−226.430
Moderate nutrient constraint	435.663⁎⁎⁎	
Severe nutrient constraint	−294.136	
North east	−348.000	
North west	−583.819⁎⁎⁎	
2013	−153.039	−49.513
Constant	900.034	522.137
Number of observations	1084	1084
R-squared	0.316	0.751

Source: Authors’ estimations from the LSMS-ISA data.

⁎ Significant at 10 percent.

⁎⁎ Significant at 5 percent.

⁎⁎⁎ Significant at 1 percent.

+ Significant at 15% or less.

**Table 4:** MPPs and APPs of applied nitrogen.

	MPP of applied nitrogen	APP of applied nitrogen
2010	7.75	7.66
2012	7.71	7.56

Source: Authors’ estimations from the production function estimates.

**Table 5:** Profitability of fertilizer use in the cereal-root crop farming system in Nigeria at current acquisition costs (assuming risk neutrality of farmers).

	2010	2012
Proportion of plots on which expanding fertilizer use can increase income (MVCR ⩾ 1)	0.49	0.53
Proportion of plots on which the net benefit from fertilizer use is positive (AVCR ⩾ 1)	0.51	0.56

Source: Authors’ estimations from the LSMS-ISA data.

**Table 6:** Transportation costs and the profitability of fertilizer use.

Year	Full acquisition cost	Fertilizer available in the village
*Proportion of maize plots for which expanding fertilizer use is profitable for a risk neutral farmer (MVCR ⩾ 1)*		
2010	0.49	0.70
2012	0.53	0.86

*Proportion of maize plots for which net benefit from fertilizer use is positive for a risk neutral farmer (AVCR ⩾ 1)*		
2010	0.51	0.86
2012	0.56	0.88

Source: Authors’ estimations from the LSMS-ISA data. Results obtained from a simulation of fertilizer profitability with and without transportation cost.

**Table 7:** A comparison of actual and expected profit maximizing nitrogen application rates for maize in Nigeria.

	Percentage of plots using fertilizer	Mean of applied nitrogen (kg/ha)	Percentage of plots currently using fertilizer that could gain from using more fertilizer at acquisition cost	Percentage of plots currently using fertilizer on which fertilizer use is beyond optimal
2010	64	72	41	23
2012	67	67	54	13

Source: Authors’ estimations from the LSMS-ISA data.

## Appendix A

See Table A1.

## Footnotes

1 Liverpool-Tasie (2016) is the only paper found for rice in Nigeria, while Sheahan et al. (2013) and Xu et al. (2009) are examples for maize in Kenya and Zambia respectively.

2 One other crop apart from maize is being grown on about half of our maize plots while over 30% grow more than two crops.

3 Farmers use different types of fertilizers on their plots, which have different nutrient contents. Thus, rather than consider all inorganic fertilizer to be the same, we isolate the nutrient component of the applied fertilizer. The two major fertilizers used in Nigeria are NPK and Urea. NPK typically has either about 27% nitrogen, 13% phosphorus and 13% potassium or 15% of each of the three nutrients, while urea is about 46% nitrogen. For this analysis, we multiply those percentages by the total amount of each fertilizer applied to the maize plot to arrive at the total quantity of applied nutrients.

4 Nitrogen has been shown to be a key constraint to cereal production in Nigeria (Ezui et al., 2010) and at least accounting for applied phosphorus reduces omitted variable bias.

5 We run the CRE and FE and get consistent results. Because of its strong assumption about the distribution of the unobserved household characteristic, we have chosen to present and focus on the fixed effects model in this analysis.

6 The coefficients on applied nitrogen are actually slightly lower in the household level analysis. Since this indicates that our results based on the plot level analysis might be a slight overestimate strengthening the papers argument about the limited profitability of fertilizer use (which increases with the yield response of nitrogen), we proceeded to use the fixed effects results at the plot level.

7 This follows Sheahan and Barrett (2014) as 700 kg/hectare represents an upper-bound limit associated with inorganic fertilizer use in the United States under irrigated corn conditions.

8 For this same reason, we are not able to explore other dimensions of nitrogen application as the likely role labor availability plays in the effectiveness and profitability of nitrogen application.

9 We calculate maize yield as the maize output per area allocated to maize rather than total plot area. Concerns about attenuation bias due to measurement error are reduced by the consistency of the household level regression. The main conclusions from a specification using area allocated to maize are upheld when total plot area is used.

10 We follow Sheahan and Barrett (2014) to use this definition but recognize that this might be an overestimation (for the purposes of capturing tenure as an indicator of likely willingness to make various agricultural investments) as in many communities in Nigeria where land is distributed by the community, this occurs yearly with no guarantee of the same plot being assigned yearly.

11 This assumes the nutrient content of NPK fertilizer is 27:13:13 while that of Urea is 46% of the fertilizer quantity.

12 We also tried to interact the applied nitrogen variable with irrigation but were unable to due to collinearity problems.

13 Whether a household member received advice on agricultural information (likely endogenous) was also considered and found not to be significant.

14 We run specifications that include a control for whether a leguminous plant is grown on the maize plot, but this did not yield significant results and was highly correlated with the number of crops grown. Thus we focused on the number of crops grown. We also tried interacting the number of crops grown on the plot with applied nitrogen but these were not significant; probably partly due to multicollinearity.

15 Local government authorities are administrative units below the state. There are 776 LGAs across Nigeria’s 36 states.

16 We replace any transportation costs (after winsorizing) that exceed N700 with N700 to exclude the effect of transportation costs for trucks and pickups. However anecdotal evidence from extension agents in rural Nigeria indicates that transportation cost even by motorcycle ranges between N200 and N2,000, depending on many factors such as the region of the country, the season, and quality of the road. Thus capping at N700 might be underestimating the true cost of a trip. The amount purchased per trip is about 100 kgs of fertilizer which is equivalent to about 23 kgs of nitrogen nutrient.

17 It can be argued that using the average cost per kg purchased does not account for the fixed costs associated with purchasing the input or economies of scale from bulk purchases, we do not consider this problematic since our applied costs are those actually paid by the farmer. Furthermore we explored the case when the transportation cost per kg applied to the market price of fertilizer for each farmer was their expressed transportation cost divided by 50 kgs (the typical size of a fertilizer bag sold in the market in Nigeria) and our main findings about high transport costs are maintained.

18 We also explore using the average transportation cost per kg for a farmers community (enumeration area) rather than his own particular average cost and the main study findings are maintained.

19 While CIF prices in Nigeria were only about $6/ton higher than US prices, retail prices in urban Nigeria were almost double due to importer marketing costs and margin, port services, finance costs; and domestic transportation cost.

20 Our dataset does not have information about whether farmers received subsidized fertilizer or not. The only indication is the response to a question on if some fertilizer applied on the plot was obtained from the government and only about 3% of our sample responds affirmatively to this. There is a question about the source of fertilizer and we consider farmers who purchased fertilizer from government as a proxy for participating in the government program, as the Nigerian government is not typically involved in the sale of fertilizer.

21 For each plot the optimal level is calculated and compared to how much was actually applied and averaged across plots. Given that optimal rates can include decimals, there are no plots on which the actual amount applied equals the optimal amount. There are less than 5% of plots on which the gap is less than 1 kg of applied nitrogen.

22 A full scale profitability would be necessary to make this claim as fertilizer use has other dimensions such as increased labor demand for application and consequent weeding and this has not been taken into account yet.
